# Investigation of Word Recognition Methods for Japanese Fingerspelling Using Large Language Models

**DOI:** 10.3390/s26196044

**Published:** 2026-09-24

**Authors:** Yousun Kang, Renta Kuzuyama, Naoto Tsuta, Ryota Murai, Duk Shin

**Affiliations:** 1 Faculty of Engineering, Tokyo Polytechnic University, Atsugi 243-0297, Japan; d.shin@t-kougei.ac.jp; 2 Graduate School of Engineering, Tokyo Polytechnic University, Atsugi 243-0297, Japan; m2663003@st.t-kougei.ac.jp (R.K.); m2563005@st.t-kougei.ac.jp (R.M.); 3 Department of Engineering, Tokyo Polytechnic University, Atsugi 243-0297, Japan

**Keywords:** Japanese fingerspelling recognition, large language models, vision-based sensing, visual-language integration

## Abstract

The development of automatic fingerspelling recognition systems is critical for facilitating seamless communication for the deaf and hard-of-hearing community. Historically, recognition technologies relied on wearable sensors, such as data gloves, inertial measurement units (IMUs), and surface electromyography (sEMG), which provided stable kinematic data but suffered from high hardware costs and user discomfort. Non-invasive vision-based sensing has emerged to eliminate wearable overhead but is subject to severe physical bottlenecks: continuous high-speed movements inevitably introduce motion blur and self-occlusion, drastically degrading tracking accuracy. To address these visual sensing constraints, we investigate a visual-language integration framework that combines dynamic visual representations derived from a single RGB video stream with the lexical knowledge of Large Language Models (LLMs), allowing the language model to compensate for uncertainty or errors in the visual representation. Our approach employs a robust visual encoder—comprising a Temporal Convolutional Network (TCN) and a Transformer—to extract and integrate dynamic transition features from 3D skeletal coordinates. Rather than propose a new LLM architecture, this study focuses on how visual information should be represented and transferred to an LLM by comparing discrete-text, probability-level, and latent-feature-level integration strategies under the same experimental conditions. Specifically, we systematically evaluated three integration strategies: Text Input (TI), Projector Probabilities Input (PPI), and Projector Transformer Features Input (PTFI). Experimental results on a 28-word closed-set evaluation show that while the word-level exact-match accuracy (WA) of the visual encoder alone was limited to 11.66% with continuous signing, our LLM-integrated architecture elevated the WA to a maximum of 87.73%. Crucially, the PTFI approach successfully suppressed character-level degradation by grounding linguistic reasoning in latent Transformer-based visual evidence. These results suggest that integrating LLM-based linguistic reasoning with dynamic visual features is a promising approach for closed-set continuous fingerspelling recognition under controlled experimental conditions.

## 1. Introduction

Facilitating seamless communication between the more than 70 million deaf and hard-of-hearing individuals worldwide and those with normal hearing remains a critical and persistent social challenge [1,2]. Sign language serves as the primary modality for daily communication, and within this system, fingerspelling plays an indispensable role, particularly in accurately conveying proper nouns, technical terminology, and newly coined terms [3,4]. However, because the vast majority of hearing individuals cannot visually recognize fingerspelling, there is an urgent need for automated systems that can translate these movements into accessible text formats.

Historically, sign language recognition (SLR) has transitioned from early physical sensor-based methods to sophisticated video-based deep learning frameworks [2,5]. Early research focused on spatial feature extraction using convolutional neural networks (CNNs) and temporal modeling via long short-term memory (LSTM), recurrent neural networks (RNNs), or temporal convolutional networks (TCNs) [6,7]. As highlighted in recent reviews, the field has progressed to complex spatiotemporal analysis [1,2,5]. More recently, Transformer-based architectures have emerged as cornerstones for state-of-the-art SLR, demonstrating superior efficacy in modeling long-range dependencies [8,9]. To further improve performance, techniques such as batch normalization [10,11], supervised contrastive learning (SupCon) [12], and connectionist temporal classification (CTC) [7] have been integrated into the pipelines.

A transformative paradigm shift is currently under way with the integration of large language models (LLMs) into SLR [13,14]. Modern research is moving toward gloss-free translation, leveraging the semantic knowledge of LLMs to bypass manual annotations [14,15]. Frameworks such as SignLLM [13] and Sign2GPT [14] have demonstrated that sign representations can be aligned with pretrained language models to improve gloss-free sign language translation. Furthermore, advancements in visual instruction tuning [15,16] and contextual embeddings such as SCOPE (Sign Language Contextual Processing with Embedding from LLMs) emphasize the importance of incorporating dialogue history and linguistic context into sign language understanding [17,18]. Related LLM-based approaches have also shown promise for low-resource sign language translation scenarios, particularly when external lexical descriptions or in-context examples are available [19].

However, the integration of LLMs into SLR does not by itself resolve the core problem of visual grounding. In many existing LLM-based SLR pipelines, visual information is first converted into glosses, discrete tokens, or highly compressed feature vectors before being interpreted by the language model. This design allows the LLM to exploit lexical and contextual priors, but it also creates a critical interface problem: once fine-grained hand-shape, motion, and uncertainty information has been discarded or corrupted by the upstream visual encoder, the LLM can only perform post hoc lexical correction. In such cases, the model may overrely on plausible linguistic patterns rather than on the actual signed evidence. Therefore, a key unresolved question is how visual information should be represented and injected into the LLM so that linguistic reasoning remains grounded in the observed hand motion.

This problem is particularly critical for continuous fingerspelling. Unlike sentence-level sign translation, fingerspelling requires character-by-character recovery from rapid hand-shape transitions, where motion blur, self-occlusion, and ambiguous boundaries between adjacent characters can accumulate into severe word-level errors [20,21]. In Japanese fingerspelling, the challenge is further amplified by 76 distinct character classes, including visually similar hand shapes and dynamic variants such as voiced and semi-voiced sounds [3,22]. These characteristics make simple text-level correction insufficient and motivate a visual-language integration strategy that grounds the LLM not only in a decoded character string but also in soft class probabilities and latent spatiotemporal visual features.

Continuous fingerspelling recognition has also been extensively studied in American Sign Language (ASL), notably through the ChicagoFSWild and ChicagoFSWild+ benchmarks and subsequent work on in-the-wild sequence recognition [23,24,25]. In contrast, the present study evaluates a controlled 28-word closed-set Japanese fingerspelling task and does not aim to demonstrate signer-independent or in-the-wild generalization. Instead, our focus is on how visual representations at different abstraction levels are transferred to a frozen LLM through TI, PPI, and PTFI.

Therefore, in this study, we investigate a visual-language integration framework that leverages LLMs such as Gemma-3 [26] and Qwen-3 [27] for continuous Japanese fingerspelling word recognition. We utilize the public ub-MOJI dataset [4] together with additional non-public recordings for the single-character recognition stage, while the word-level experiments use only recordings from the 18 participants included in the public dataset. We implement a visual encoder based on TCN and Transformer architectures [6,8]. Using MediaPipe for hand landmark extraction [28], we integrate static angle features with dynamic trajectory and velocity cues.

Although recent LLM-based sign language studies, including SignLLM, Sign2GPT, and LLaVA-SLT, have explored the alignment of sign or visual representations with pretrained language models, the main contribution of this study is not the use of an LLM itself. Instead, we systematically compare three interface representations between a visual fingerspelling encoder and an LLM: decoded text; soft class-probability distributions; and continuous latent Transformer features, corresponding to Text Input (TI), Projector Probabilities Input (PPI), and Projector Transformer Features Input (PTFI), respectively. By evaluating these three strategies with the same visual encoder, dataset split, training protocol, and LLM backbones, this study isolates how the abstraction level of visual information affects word-level recognition accuracy and character-level structural fidelity.

The base LLMs are loaded with four-bit quantized frozen weights and are not fine-tuned; for the projector-based PPI and PTFI strategies, only the lightweight projector that maps visual representations into the LLM embedding space is trained. Our results show that the proposed PTFI strategy significantly improves word-level exact-match accuracy (WA) to 87.73%, demonstrating the effectiveness of continuous latent visual grounding for closed-set continuous Japanese fingerspelling recognition.

## 2. Materials and Methods

The Japanese fingerspelling word recognition system proposed in this study consists of three sequential stages: (1) feature extraction from video, (2) single-character recognition using a visual encoder, and (3) word recognition using an LLM. This architecture is designed to overcome the inherent physical limitations of visual sensing by leveraging high-level linguistic intelligence.

### 2.1. Feature Extraction and Representation

The MediaPipe Hand Landmarker [28] extracts the 21-point hand skeleton from each video frame, as illustrated in Figure 1. Let $p_{n}$ represent the *n*th joint coordinate and $p_{0}$ denote the wrist coordinate.

Trajectory and velocity features are calculated as dynamic transition information and integrated with the static angle feature (Angle) proposed by Kondo et al. [3]. These combined elements constitute the input data for the visual encoder, enabling the system to capture both the static shape and the temporal flow of fingerspelling movements.

The angular features belong to three types: finger flexion (15 dimensions), interdigital angle (4 dimensions), and hand orientation (30 dimensions). The angle between two vectors is calculated as shown in Equation (1).

(1) $$\theta =\arccos\left(\frac{a\cdot b}{∥a∥∥b∥}\right)$$

Finger flexion represents the degree of bending and is defined as the angle between adjacent vectors within the same finger. For three consecutive landmarks on a finger, these vectors are calculated using Equation (2):

(2) $$v_{1}=p_{i}-p_{j},\;v_{2}=p_{k}-p_{j},$$

where $p_{i}$, $p_{j}$, and $p_{k}$ denote the coordinates of the respective joint landmarks.

Interdigital angle represents the spread or closure of the fingers and is defined as the angle between the bases of adjacent fingers. For a wrist landmark and the base landmarks of two adjacent fingers, the corresponding vectors are defined in Equation (3) as follows:

(3) $$v_{1}=p_{\mathrm{mcp}1}-p_{0},\;v_{2}=p_{\mathrm{mcp}2}-p_{0}.$$

The third angular component is computed from the orientation of wrist-to-landmark vectors relative to fixed spatial reference axes. In generic form, the two vectors used in the cosine angle calculation are defined in Equation (4):

(4) $$v_{1}=p_{\mathrm{target}}-p_{0},\;v_{2}=e_{\mathrm{axis}},$$

where $p_{\mathrm{target}}$ denotes a selected finger landmark and $e_{\mathrm{axis}}$ denotes a fixed Cartesian reference-axis vector.

Following the cosine-based orientation formulation used in our previous work [3], we calculate orientation features from vectors originating at the wrist landmark $p_{0}$. Ten target landmarks are selected: $p_{2},p_{4},p_{5},p_{8},p_{9},p_{12},p_{13},p_{16},p_{17}$, and $p_{20}$, corresponding to the MCP joint and fingertip of each of the five fingers. For each target landmark $p_{j}$, the wrist-to-landmark vector is defined as

(5) $$v_{j}=p_{j}-p_{0}.$$

The orientation of each vector is then calculated with respect to the three fixed Cartesian reference axes,

(6) $$e_{x}=\left(1,0,0\right),\;e_{y}=\left(0,1,0\right),\;e_{z}=\left(0,0,1\right),$$

using the cosine-angle formulation in Equation (1). Thus, three orientation values are obtained for each of the ten selected landmarks, resulting in a 30-dimensional hand-orientation feature vector (10×3=30).

Compared with our previous work [3], the present study limits the orientation targets to the ten MCP and fingertip landmarks listed above. Combined with the 15 finger-flexion and 4 interdigital-angle features, the resulting 30 orientation features form a 49-dimensional angle feature vector, denoted as $A_{t}$, for each frame.

To capture the dynamic nature of fingerspelling, we complement these static shape features with trajectory and velocity information. Trajectory displacement ($T_{t}$) is defined as the displacement vector of the wrist coordinate at frame *t* relative to its initial position at the start of the motion, as shown in Equation (7). This captures the overall spatial path of the hand movement.

(7) $$T_{t}=p_{0}^{\left(t\right)}-p_{0}^{\left(1\right)}$$

Velocity displacement ($V_{t}$) represents the frame-to-frame wrist displacement between the current and previous frames, serving as a velocity cue that captures the speed and rhythm of hand transitions.

(8) $$V_{t}=p_{0}^{\left(t\right)}-p_{0}^{\left(t-1\right)}$$

It should be noted that both trajectory displacement ($T_{t}$) and velocity displacement ($V_{t}$) were computed from the original wrist coordinates before wrist-centered normalization. The original wrist trajectory was retained separately for computing $T_{t}$ and $V_{t}$, whereas the wrist-centered normalized coordinates were used for calculating the angular feature ($A_{t}$). Therefore, the global wrist displacement information was not removed by the normalization process.

To effectively capture the spatial and temporal nuances of Japanese fingerspelling, we extract 3D hand landmarks using MediaPipe [28] and define three raw feature components: angle feature ($A_{t}\in R^{49}$), trajectory displacement ($T_{t}\in R^{3}$), and velocity displacement ($V_{t}\in R^{3}$). To compare the contribution of trajectory and velocity information across recognition stages, these components are combined into three distinct feature sets utilized throughout our experiments: TA, VA, and TVA.

As defined in Equations (9)–(11), these integrated feature sets allow the visual encoder to simultaneously process the static configuration of the hand and its complex spatiotemporal dynamics:

- **TA (trajectory-augmented angle)** combines the directional movement of the hand’s path ($T_{t}$) with its internal geometric configuration ($A_{t}$), focusing on the spatial path of the gesture.
- **VA (velocity-augmented angle)** integrates the frame-to-frame wrist displacement, referred to as velocity displacement ($V_{t}$), with the static joint angles ($A_{t}$), characterizing dynamic hand transitions.
- **TVA (trajectory- and velocity-augmented angle)** provides a comprehensive representation by fusing all three components—trajectory displacement ($T_{t}$), velocity displacement ($V_{t}$), and static joint-angle configuration ($A_{t}$).

The resulting input feature vectors $f_{t}$ have a dimension of 52 or 55, depending on the specific feature set:

(9) $$\mathrm{TA}:\;f_{t}={\left[T_{t},A_{t}\right]}^{⊤}\in R^{52}$$

(10) $$\mathrm{VA}:\;f_{t}={\left[V_{t},A_{t}\right]}^{⊤}\in R^{52}$$

(11) $$\mathrm{TVA}:\;f_{t}={\left[T_{t},V_{t},A_{t}\right]}^{⊤}\in R^{55}$$

This hierarchical representation ensures that the model maintains robustness against variations in global hand orientation while capturing the essential temporal transitions between fingerspelling characters.

### 2.2. Single-Character Recognition Using a Visual Encoder

As a visual encoder for character recognition based on extracted time-series features, we adopted an architecture that combines a TCN [6], which captures local temporal dependencies, with a Transformer [8], which aggregates global temporal context. In this framework, batch normalization [10,11] is applied to each layer of the TCN to stabilize training and improve generalization performance. To enhance the model’s robustness against common challenges such as self-occlusion and motion blur, the TCN is first pretrained independently using SupCon [12].

During the primary training phase, which integrates both the TCN and Transformer components, online data augmentation techniques—including the addition of minor Gaussian noise to the features in each epoch and feature dropout—are applied. The overall architecture of the model is illustrated in Figure 2.

### 2.3. A Word Recognition Approach Using an LLM

To recognize the final word sequence based on the temporal inference results from the visual encoder, we integrate a downstream LLM. This study constructs and compares three distinct integration methods—TI, PPI, and PTFI—which vary in the level of abstraction of the representations passed from the visual domain to the language model.

The first method, TI, connects the systems via discrete text. We apply a sliding window to the visual encoder’s output (logits) and convert them into a discrete hiragana string as a prompt for the LLM. To mitigate noise during this conversion, we implement a heuristic decoding process rather than a simple argmax sequence. Specifically, characters exceeding a defined probability threshold are extracted as valid candidates, while others are treated as blanks. Following the decoding logic of CTC [7], consecutive identical characters are compressed, forming a word string only when separated by blanks. The LLM then identifies the most probable word by leveraging its internal lexical knowledge to compensate for noise or missing characters.

The remaining two methods input the visual encoder’s feature representations directly as continuous-valued vectors. While the TI method loses information regarding visual ambiguity, such as the probabilities of secondary candidates, these proposed methods preserve such nuances. We developed a mechanism to feed data directly into the LLM’s embedding space via a projector layer composed of a multi-layer perceptron (MLP), following established visual instruction tuning paradigms [16].

For the PPI method, the input to the projector is the 76-dimensional softmax probability vector produced by the visual encoder. For the PTFI method, the input is the 256-dimensional latent feature vector obtained from the final Transformer layer of the visual encoder. Each projector is implemented as an MLP-based adapter consisting of layer normalization, a linear layer, GELU activation, dropout, a second linear layer, and a final layer normalization. The hidden dimension and output dimension of the projector are both set to 2560, corresponding to the token embedding dimension of the LLM. The projected visual embedding is incorporated into the LLM input embedding sequence as a soft visual prompt, together with the text instruction prompt. During training, only the projector parameters are updated using the generation loss for the target word, while both the visual encoder and the LLM remain frozen. To enhance robustness, we apply on-the-fly data augmentation, including Gaussian noise and feature dropout, as described in Section 2.2. This allows the projected visual representations to function as trainable soft prompts for the frozen LLM.

The integration pipeline between the word recognition system and the LLM, along with the detailed structure of the projector layer, is illustrated in Figure 3.

### 2.4. Dataset and Preprocessing

The dataset used in this study comprises the publicly available ub-MOJI data [4] and additional non-public recordings collected as part of the same Japanese fingerspelling data collection project. All 23 participants were external volunteers learning sign language in a sign language class, and none were members of the laboratory. The data are organized into two subsets: single-character data, encompassing 76 classes with 1605 samples, and word-level data, comprising 28 words with 326 total samples.

The sample distributions for both character-level and word-level datasets are illustrated in Figure 4 and Figure 5. The word data is specifically organized into kana syllabary sequences, meaningful words, and nonsense strings to evaluate recognition performance across various linguistic contexts. The kana syllabary sequence group was included to ensure that the word-level dataset covered the full set of 76 single-character classes used in the isolated character recognition task. Thus, the 28 word-level classes collectively contain all 76 character classes, although the evaluation itself remains a 28-word closed-set task.

To prepare the input for the visual encoder, 3D hand skeleton coordinates are extracted frame by frame via MediaPipe. These coordinates are normalized relative to the wrist position ($p_{0}$) and scaled by the hand’s dimensions to ensure invariance to camera distance and individual physical differences. Data quality is further maintained by filtering out frames with low detection confidence scores. Based on these refined coordinates, the three feature types (TA, VA, and TVA) defined in Section 2.1 are calculated. This wrist-centered normalization was applied to the landmark coordinates used for angular feature extraction. The original wrist coordinates were retained separately and used to compute the trajectory and velocity displacement features described in Section 2.1.

The system’s training and evaluation proceed in two sequential stages: single-character recognition by the visual encoder followed by word-level recognition through LLM integration. For the character recognition model, the dataset is partitioned into 80% for training and 20% for testing. Since most single-character videos span approximately one second at 30 fps, the target input length is set to 30 frames. To accommodate variations in original video lengths, all sequence data are initially interpolated to a uniform length of 60 frames.

Generalization performance is enhanced by applying on-the-fly data augmentation, including random noise injection and magnitude warping, to the training set. To address class imbalances, oversampling is performed for minority classes. This involves randomly extracting pre-augmented 60-frame data and generating new samples, effectively equalizing the distribution across all 76 classes.

A subsequent resampling process reduces the 60-frame sequences to the final 30-frame inputs. A random cropping strategy is employed for the training set to improve temporal robustness, while the test set utilizes a center cropping strategy to capture the core movement. This 30-frame standard also serves as the window size for the sliding window in the word recognition phase. Finally, zero padding is applied to unify the maximum sequence length to 400 frames for batch processing compatibility, with the processed data stored in HDF5 format.

For word-level recognition, the 326 word-level video samples were split into training and test sets at a ratio of 5:5, resulting in 163 training samples and 163 test samples. The split was performed before data augmentation using a stratified strategy to preserve the word-class distribution. Ten percent of the training samples were used as a validation set for model selection and early stopping. The test set was not used during projector training, hyperparameter tuning, or data augmentation. Therefore, all WA and CER values reported in Tables 4–6 were computed on held-out word-level test videos. This evaluation was conducted in a closed-set context: all word classes appearing in the test set were included in the training vocabulary, but the test videos themselves were unseen during training.

The complete data collection involved 23 participants, all of whom were external volunteers learning sign language in a sign language class. Of these participants, 18 provided permission for public data release and are included in the publicly available ub-MOJI dataset, whereas recordings from five participants are not publicly released because permission for public distribution was not obtained. The non-public recordings were used only in the single-character recognition experiments. The word-level dataset used for LLM training and evaluation consists exclusively of 326 public ub-MOJI videos from the 18 publicly released participants; therefore, no non-public recordings were included in the word-level training, validation, or test sets.

The public ub-MOJI dataset does not currently provide an official train/test split. Therefore, we defined the split used in this study. The word-level split was performed at the sample level and was not signer-independent. All 18 signers appeared in both the training and test sets through recordings of different words; therefore, no signer was assigned exclusively to either the training set or the test set. For 16 of the 18 signers, each signer–word pair was represented by a single video. For the remaining two signers, repeated recordings were available for selected kana syllabary sequences, with up to three videos for the same signer–word pair. In some cases, all repeated recordings of the same signer–word pair were assigned to either the training or the test set after the sample-level split. Accordingly, the reported results should be interpreted as a 28-word closed-set evaluation under a signer-overlapping sample-level split, rather than as evidence of generalization to unseen signers or open-vocabulary real-world fingerspelling recognition.

### 2.5. Evaluation Metrics

The performance of the word recognition system integrated with the LLMs is quantitatively evaluated using two primary metrics: word-level exact-match accuracy (WA) and character error rate (CER). In this study, WA denotes the percentage of samples for which the entire predicted word string exactly matches the ground-truth sequence; it is not defined as 1−WER. This metric serves as the principal measure for assessing the effectiveness of the character interpolation and linguistic correction performed by the language model.

CER measures recognition precision at the character level based on the Levenshtein distance. It is calculated by dividing the sum of substitutions (*S*), deletions (*D*), and insertions (*I*) required to transform the inferred string into the ground truth by the total number of characters in the ground-truth string (*N*). This relationship is defined in Equation (12):

(12) $$\mathrm{CER}=\frac{S+D+I}{N}$$

A lower CER signifies higher character-level fidelity in the recognition process, reflecting the system’s ability to maintain structural accuracy even when perfect word-level matches are not achieved.

### 2.6. Implementation Details and Experimental Setup

Hardware and software configurations for our experiments consist of a local workstation equipped with an Intel Core i7-12700F CPU, 64 GB of RAM, and an NVIDIA GeForce RTX 3080 GPU with 10 GB VRAM. The system operates on Windows 11 Home via Anaconda, utilizing Python 3.12.9, PyTorch 2.9.0, CUDA 12.6, and cuDNN 9.10.2. Model components are implemented using MediaPipe 0.10.21 for hand extraction, while Hugging Face Transformers 4.57.0 and bitsandbytes 0.48.2 facilitate LLM integration and 4-bit quantization.

Optimization of the visual encoder follows a two-stage protocol. Initial pre-training of the TCN employs SupCon [12], after which a Transformer is integrated for transfer learning on the classification task. Hyperparameter tuning is conducted via Bayesian optimization within the Optuna framework [29], identifying optimal configurations for learning rates, optimizers (AdamW and SGD), and augmentation parameters.

The word recognition pipeline utilizes Gemma-3-4B-it and Qwen-3-4B as base language models. To address hardware constraints, the base LLMs were loaded with 4-bit quantization using bitsandbytes, while all LLM parameters were kept frozen. No LoRA adapters were introduced or trained. This configuration enables stable training of the projector layer while significantly reducing VRAM requirements. The training process exclusively optimizes the projector layer using cross-entropy loss for target word generation, thereby preserving the pretrained linguistic knowledge of the underlying LLMs, while the TI method utilizes the LLM as a JSON-based error correction engine, the PPI and PTFI methods inject projected visual feature embeddings into the LLM input embedding sequence as soft prompts. Final outputs are standardized to Japanese hiragana through a post-processing stage using the pykakasi library, which converts kanji or katakana and filters out non-Japanese symbols.

Table 1 summarizes the optimized system hyperparameters identified through the tuning process.

## 3. Results

The performance of the proposed framework is evaluated through comparative experiments focusing on feature types and LLM integration strategies. All evaluations utilize Gemma-3-4B-it [26] and Qwen-3-4B [27] to ensure a comprehensive assessment of linguistic interpolation capabilities.

### 3.1. Standalone Performance of the Visual Encoder

Initial experiments assess the recognition accuracy of the standalone visual encoder—comprising the TCN and Transformer architecture [6,8]—on isolated single-character fingerspelling before its integration with the LLM. This baseline evaluation is critical to understanding the quality of the visual features serving as the system’s foundation. Table 2 summarizes the single-character recognition accuracy across the test dataset.

The results indicate that, for isolated single-character recognition, the TVA feature set, which combines trajectory displacement, velocity displacement, and static angle features, achieved the highest accuracy of 86.44%, compared with 82.55% for TA and 82.87% for VA. This result shows that combining trajectory and velocity cues is beneficial for the isolated-character recognition task. However, this does not imply that TVA is universally optimal for downstream continuous word recognition, as the relative effectiveness of the feature sets varies depending on the recognition stage and evaluation metric.

Notably, this accuracy appears lower than the 99% reported in prior work by Kondo et al. [3], which utilized only angular features. However, this discrepancy is not a reflection of model deficiency but stems from a deliberate increase in task complexity. Our study intentionally expands the recognition targets from 46 classes to 76 classes, incorporating dynamic expressions such as voiced and semi-voiced sounds [3].

This expansion requires the model to discriminate between “similar finger signs” that share identical hand shapes but differ in their dynamic execution, a significantly more challenging objective. Furthermore, whereas previous studies often focused on “textbook-style” data, our dataset includes everyday variations and continuous transitions, which naturally introduce motion blur and self-occlusion. Achieving 86.44% under these rigorous conditions demonstrates that the encoder successfully captures motion features, providing a robust visual foundation for subsequent LLM integration.

To better understand the behavior of the visual encoder, we additionally analyzed the per-class performance of the 76 fingerspelling classes. The complete precision, recall, F1-score, and support values for all 76 classes are provided in Supplementary Table S1 for the TVA feature set and Supplementary Table S2 for the VA feature set. Here, support indicates the number of test samples for each character class. Table 3 summarizes representative classes that were reliably recognized or remained difficult to distinguish.

The representative results show that several visually distinctive or stable classes, such as *ku, wa*, and *no*, were recognized reliably. In contrast, classes such as *nn*, *ko*, *po*, and *xyu* showed lower recall or F1-scores. In particular, some of the lowest scores were observed in low-support classes, such as *po* and *xyu*; therefore, these results should be interpreted cautiously. These character-level errors are important because they can propagate to the downstream word-level stage, especially in the TI method, where the LLM receives only a discrete decoded string.

### 3.2. Visual Baseline for Word Recognition

To establish a comparative baseline, we first evaluated the word recognition performance using only the TCN-Transformer-based visual encoder’s outputs, without linguistic interpolation from the LLM. For this task, a sliding window and a deterministic argmax operation were applied to the continuous video sequences. To generate the character strings for this baseline, the encoder’s framewise outputs underwent confidence filtering and CTC-like compression, following the protocol defined for the text-based integration method in Section 2.3.

Table 4 presents the WA and CER for each feature set under this rule-based word formation logic, evaluated on the 163 held-out word-level test videos.

Table 4 also reveals that the relative ranking of VA and TVA differs depending on the evaluation metric. TVA achieved a higher WA than VA (11.66% vs. 9.82%), whereas VA produced a lower CER (59.18% vs. 61.51%). This difference reflects the distinct characteristics of the two metrics: WA requires an exact word-level match, whereas CER measures character-level distortion in the predicted sequence. Therefore, a feature set may increase the number of exactly recognized words while still producing larger character-level errors among the remaining incorrect predictions.

The experimental results reveal a substantial performance gap between isolated character recognition and continuous word formation. Despite the visual encoder achieving 86.44% accuracy for single characters, its word-level accuracy remains capped at 11.66% under the TVA feature set. This sharp decline is attributed to the inherent visual noise in continuous gestures, including motion blur, self-occlusion, and ambiguous transitions at the “seams” between characters [1,2].

In a rule-based framework, even minor temporal misalignments or single-character misrecognitions lead to a total failure in word matching. These findings suggest that continuous fingerspelling recognition in the present controlled setting may benefit from a mechanism capable of resolving lexical ambiguity through contextual knowledge, such as the proposed LLM integration.

### 3.3. Comparison of LLM Integration Methods and Analysis of Trade-Offs

To overcome the limitations of rule-based word formation described in Section 3.2, we compared three LLM integration strategies: TI, PPI, and PTFI. The TI method uses the decoded character string from the visual encoder as discrete text input. In contrast, PPI and PTFI pass continuous-valued visual representations to the LLM through a trainable MLP projector. PPI uses the softmax probability distribution of the encoder output, whereas PTFI uses the latent feature vector obtained from the final Transformer layer and maps it into the LLM embedding space.

The word recognition performance using Gemma-3-4B-it and Qwen-3-4B, evaluated on the same 163 held-out word-level test videos, is summarized in Table 5 and Table 6, respectively. Because the contribution of lexical knowledge may differ depending on whether the target sequence is linguistically predictable, we report WA and CER separately for three word categories: kana syllabary sequences, meaningful words, and nonsense strings.

The categorywise results first show that the TI method remained weak across all three word groups. Since TI uses only the discrete decoded string from the visual encoder, the LLM has little access to the uncertainty or alternative candidates contained in the visual output. For Gemma-3-4B-it, the best TI results were 11.38% WA for kana syllabary sequences, 10.65% for meaningful words, and 12.38% for nonsense strings. Qwen-3-4B showed a similar tendency, with the best TI results remaining at 13.04%, 13.10%, and 9.96%, respectively. These results indicate that post hoc correction of a corrupted text string is insufficient, even when the target sequence is linguistically meaningful.

In contrast, both PPI and PTFI substantially improved recognition performance. For Gemma-3-4B-it, PPI with VA features achieved high WA for kana syllabary sequences and meaningful words, reaching 87.45% and 88.38%, respectively. However, its WA for nonsense strings was lower at 65.88%, and the CER remained high across all categories. This suggests that probability-level input allows the LLM to use lexical knowledge effectively, especially for linguistically predictable words, but it can still produce structurally distorted outputs when the visual representation is too compressed.

The coexistence of high WA and relatively high CER in some PPI settings can be explained by the different sensitivities of the two metrics. WA measures exact word-level matches, whereas CER reflects character-level distortion in the incorrect outputs. Therefore, high WA can coexist with relatively high CER when many samples are recognized exactly but the remaining errors involve multiple insertions, deletions, or substitutions.

The clearest effect of latent visual grounding was observed with Qwen-3-4B. Under the PTFI setting with VA features, the model achieved 88.35% WA for kana syllabary sequences, 88.69% for meaningful words, and 82.93% for nonsense strings. The corresponding CER values were 13.41%, 9.46%, and 17.93%, respectively. The strong result for meaningful words confirms that lexical knowledge helps recover visually ambiguous character sequences. At the same time, the high performance on nonsense strings shows that the improvement was not simply due to lexical guessing. Rather, PTFI appears to preserve latent visual information that remains useful even when linguistic predictability is limited.

These results support the view that the level of visual representation transferred to the LLM is critical. PPI provides useful probability-level information, but it can still overrely on lexical priors. PTFI, in contrast, gives the LLM access to richer latent visual features and better preserves character-level structure. This explains why PTFI with VA features provided the most balanced performance across WA and CER.

Taken together with the isolated-character results, these findings indicate that the optimal feature configuration is task-dependent. Although TVA achieved the highest accuracy for isolated single-character recognition, VA provided stronger performance in the best-performing LLM-integrated word-recognition settings. Thus, improved isolated-character classification accuracy does not necessarily translate directly into improved continuous word-level recognition after LLM integration. We therefore interpret TVA as the best-performing configuration for the isolated-character task, rather than as a universally preferred feature set.

## 4. Discussion

The experimental results suggest a shift in how continuous fingerspelling can be approached: moving away from deterministic visual decoding and toward LLM-assisted visual-language integration. As our evaluation shows, the TI method failed to break the 13% WA barrier across all tested configurations. This consistently low performance highlights a critical bottleneck: once a visual sequence is corrupted by spatiotemporal noise, post hoc lexical correction alone may be insufficient to reconstruct the original tokens. This limitation in capturing the fluid transitions of Japanese fingerspelling characters was previously discussed by Kondo et al. [3]. Thomas et al. [21] also noted that these rapid transitions and phonetic ambiguities make fingerspelling a primary source of translation error, despite its significant role in natural sign language.

While the Probability-based (PPI) strategy did improve accuracy by preserving “soft” visual evidence, the 53.01% CER we observed with Gemma-3 indicates that substantial character-level distortion can remain despite improved WA. This outcome aligns with concerns raised by Gong et al. [13] regarding the “messy” nature of character-level sign tokens. Without a sufficiently informative visual anchor, the LLM may overrely on linguistic priors, resulting in structurally distorted output sequences. This behavior is reflected in the relatively high CER observed in some PPI configurations despite their high WA.

In contrast, our PTFI method provided a more favorable balance between recognition accuracy (87.73% WA) and structural fidelity (12.05% CER). By injecting continuous latent Transformer features directly into the LLM embedding space, the model’s linguistic reasoning can remain grounded in the underlying visual data. This richer visual grounding may help reduce the large character-level distortions observed in some PPI settings.

Previous studies by Lee et al. [30] and Lu et al. [20] addressed visual occlusion using additional wearable sensors, such as IMUs [30] and bending sensors [20]. In contrast, our framework achieved 87.73% WA using a single RGB-derived landmark stream with LLM-assisted visual-language integration, without additional wearable sensing modalities. This suggests that LLM-assisted visual-language integration is a promising software-based approach for complementing vision-based fingerspelling recognition. Finally, the fact that the best level of visual abstraction depends on the specific LLM architecture adds an important variable to the design recipes explored in recent vision-language literature [31].

The present study should therefore be regarded as an initial controlled evaluation of how different levels of visual representation can be transferred to an LLM for Japanese fingerspelling word recognition. The database is currently being expanded to include more word classes, more samples, and a broader range of participants. Since this process requires additional recording, annotation, consent management, and quality control, the present manuscript reports results based on the curated subset available for this study. Large-scale signer-independent and open-vocabulary evaluations remain important future work.

## 5. Conclusions

This study investigated how visual information should be transferred to an LLM for Japanese fingerspelling word recognition. In a 28-word closed-set Japanese fingerspelling task, we compared three integration strategies—TI, PPI, and PTFI—under the same visual encoder, dataset split, training protocol, and LLM backbones. The visual-only baseline remained limited to 11.66% WA, whereas LLM-assisted visual-language integration substantially improved word-level recognition. The best overall configuration in the full word-level test set achieved 87.73% WA, and the categorywise analysis further showed that Qwen-3-4B with PTFI and VA features reached 88.69% WA for meaningful words and 82.93% WA for nonsense strings.

Our comparative evaluation highlighted the trade-off between visual evidence and linguistic priors. The TI method was vulnerable to errors already present in the discrete decoded string, while PPI improved WA by providing probability-level information but sometimes produced higher CER when the visual representation was too compressed. In contrast, PTFI provided the most stable balance among the evaluated strategies. By injecting continuous latent Transformer features directly into the LLM embedding space, PTFI allowed the LLM to use linguistic knowledge while still preserving character-level visual structure. This was particularly evident in the Qwen-3-4B results, where PTFI with VA features achieved the highest WA and the lowest CER across all three word categories among the evaluated settings.

These findings suggest that the choice of visual representation is a key factor in LLM-assisted fingerspelling recognition. Rather than relying only on post hoc correction of decoded text, transferring latent visual features to the LLM can provide a stronger visual anchor for linguistic reasoning. The results also indicate that the optimal level of visual abstraction may depend on the underlying LLM architecture.

Future work will focus on extending this framework to larger vocabularies and signer-independent, in-the-wild datasets in order to evaluate its generalization across diverse signers and real-world environments. We also plan to investigate real-time inference optimization and advanced visual-language integration architectures, such as cross-attention mechanisms, to further refine the interaction between visual and linguistic representations. These steps will be necessary to bridge the gap between the present closed-set controlled evaluation and future assistive communication systems for the deaf and hard-of-hearing community.

## Figures and Tables

**Figure 1:**
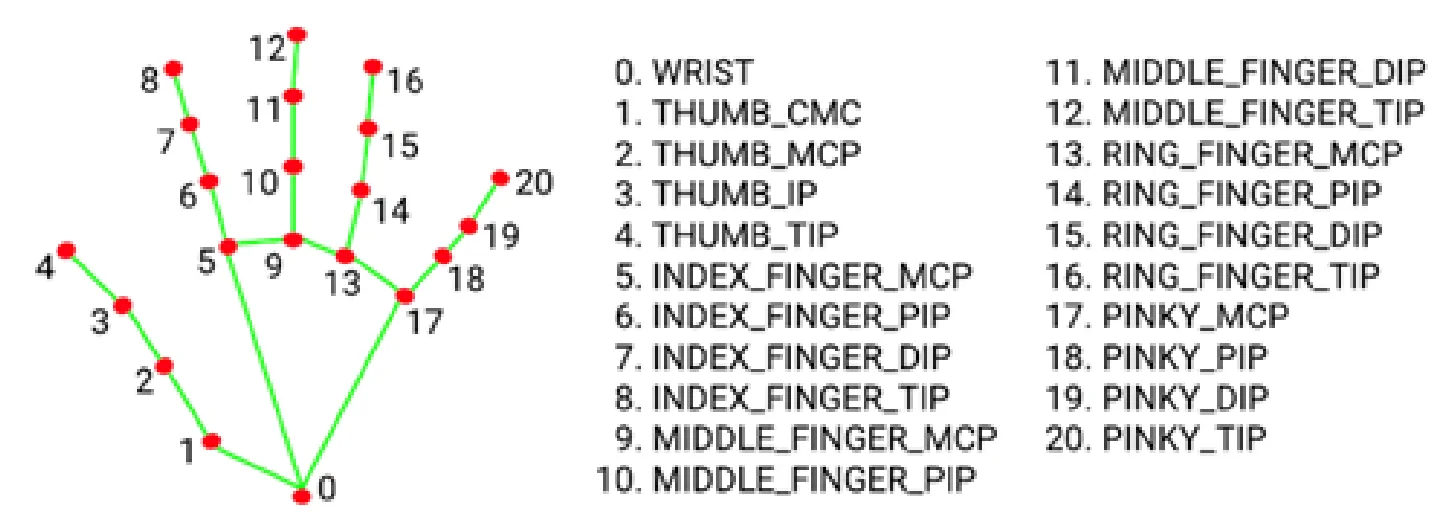
Hand skeleton coordinates obtained using MediaPipe.

**Figure 2:**
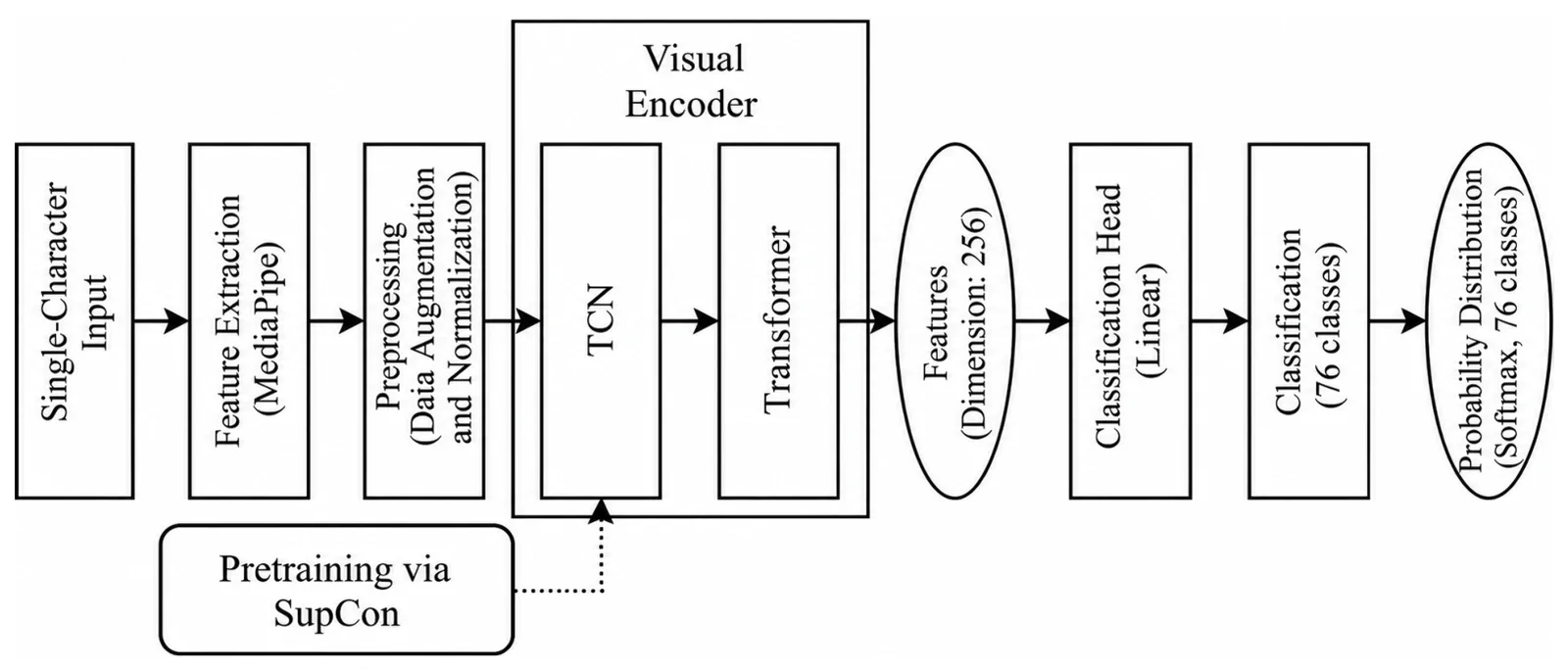
Overview of the visual encoder architecture, combining a TCN and a Transformer to capture local and global temporal dependencies.

**Figure 3:**
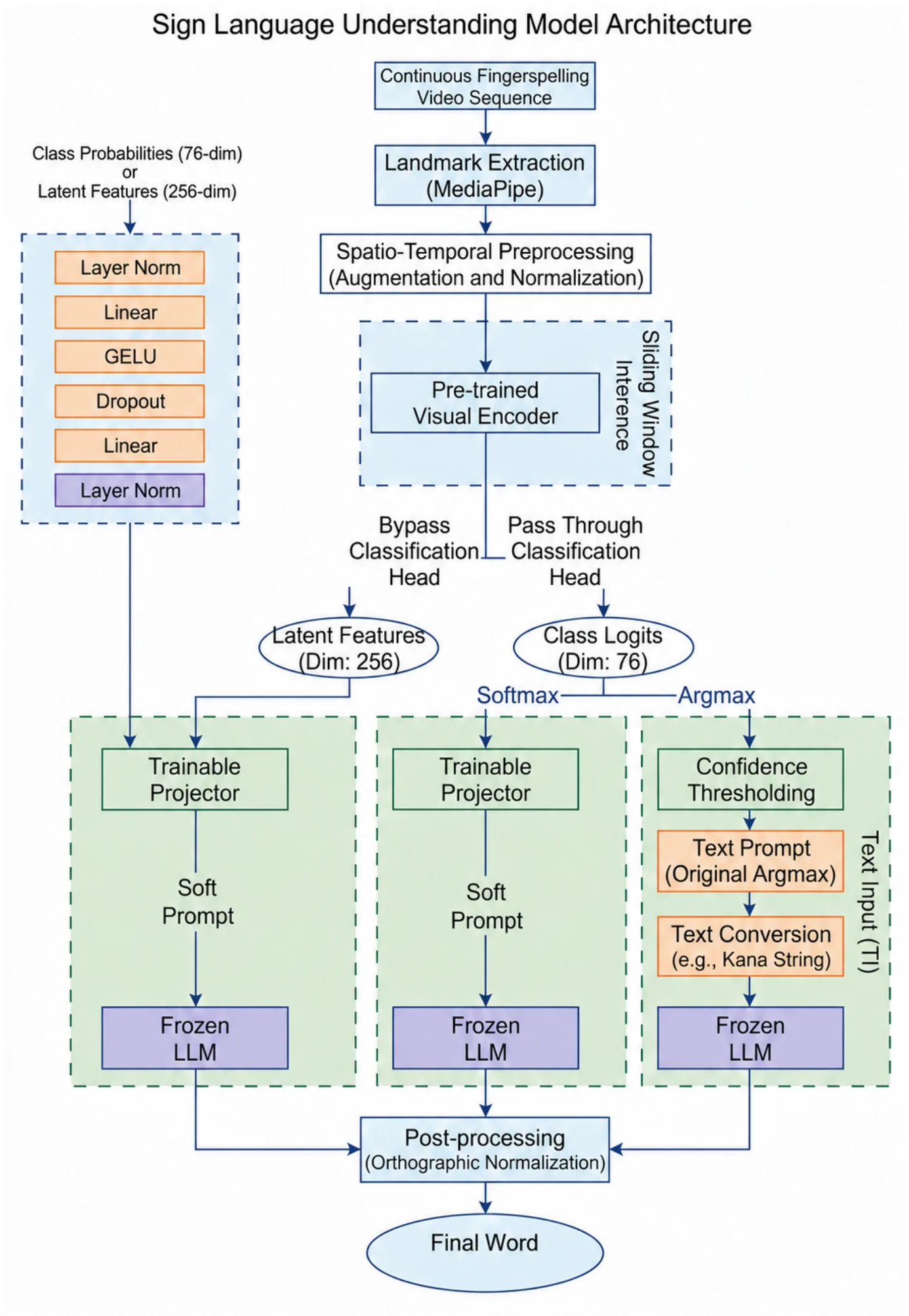
Overview of the word recognition pipeline integrating the visual encoder with the LLM and of the detailed architecture of the projector used in the continuous-valued vector input methods (PPI and PTFI). The visual encoder is pretrained on isolated single-character samples and subsequently applied to continuous fingerspelling sequences using a sliding-window strategy.

**Figure 4:**
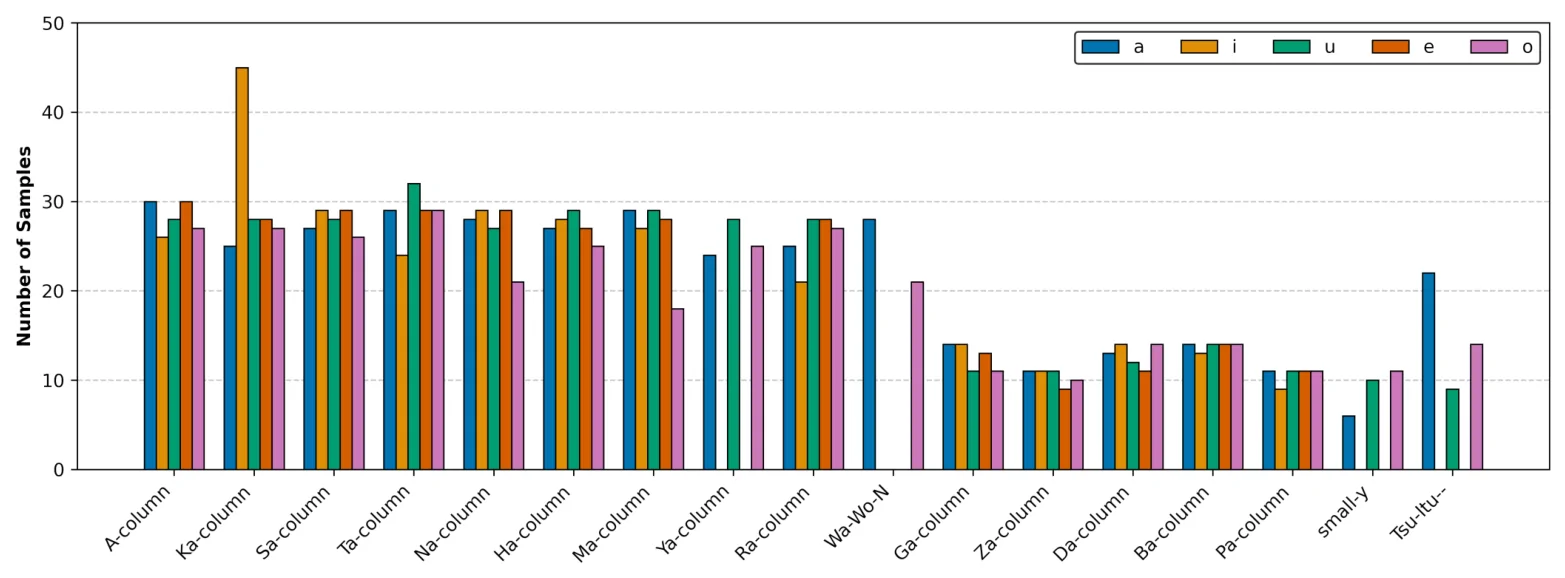
Sample distribution across 76 classes in the single-character dataset.

**Figure 5:**
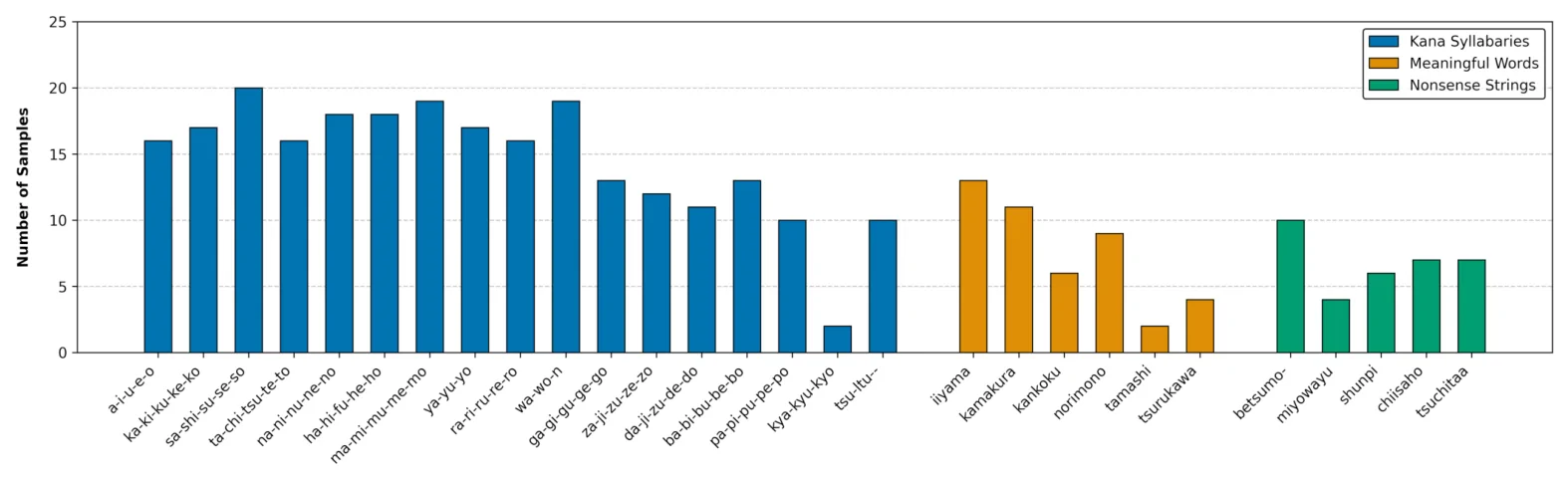
Sample distribution across different classes in the word dataset, categorized into Kana Syllabary Sequences, Meaningful Words, and Nonsense Strings.

**Table 1:** Detailed hyperparameters of the overall system.

Module/Phase	Parameter	Value
Preprocessing and	MediaPipe confidence (detection and tracking)	0.5
sliding window	Input frames (window size)/stride	30/5
	TI method confidence threshold	0.6
TCN (pretraining)	Hidden dimensions/layers/ kernel size	256/5/5
	Optimizer/learning rate/batch size	AdamW/$5.9\times 10^{-4}$/32
Transformer	Hidden dimensions/layers/heads	128/2/2
(single-char. rec.)	Optimizer/learning rate/batch size	SGD/$1.4\times 10^{-2}$/128
	Encoder weights frozen	True
LLM integration	Base LLM weight quantization	4-bit
	LLM parameters	Frozen
	LoRA adapters	Not used
	Trainable module	Projector only
Projector (PPI method)	Projector structure	2-layer MLP (dropout: 0.1)
	Learning rate/epochs/batch size	1.0 × $10^{-4}$/200/1
Projector (PTFI method)	Projector structure	2-layer MLP (dropout: 0.3)
	Learning rate/epochs/batch size	1.0 × $10^{-4}$/300/1

**Table 2:** Single-character recognition accuracy of the standalone visual encoder.

Feature Set	Included Components	Accuracy [%]
TA	Trajectory ($T_{t}$) + angle ($A_{t}$)	82.55
VA	Velocity ($V_{t}$) + angle ($A_{t}$)	82.87
TVA	Trajectory ($T_{t}$) + velocity ($V_{t}$) + angle ($A_{t}$)	86.44

**Table 3:** Representative per-class results of the visual encoder using TVA features. Support indicates the number of test samples for each class.

Class	Reason for Selection	Precision	Recall	F1-Score	Support
*ku*	Reliably recognized static class	1.000	1.000	1.000	6
*wa*	Reliably recognized static class	1.000	1.000	1.000	6
*no*	Reliably recognized motion-dependent class	1.000	1.000	1.000	4
*nn*	Dynamic or visually ambiguous class	0.667	0.500	0.571	4
*ko*	High precision but lower recall	1.000	0.500	0.667	5
*po*	Low-support semi-voiced class	0.250	0.000	0.000	2
*xyu*	Low-support small-kana class	0.500	0.000	0.000	2

**Table 4:** Word recognition performance of the visual encoder without LLM integration.

Feature Set	WA [%]	CER [%]
TA	7.36	71.37
VA	9.82	59.18
TVA	11.66	61.51

**Table 5:** Categorywise word recognition performance of integration methods using Gemma-3-4B-it.

Gemma-3-4B-it					
Method	Feature	Metrics	Kana Syllabaries	Meaningful Words	Nonsense Strings
TI	TA	WA [%]	7.35	7.81	6.12
		CER [%]	69.31	69.78	72.19
	VA	WA [%]	10.06	9.94	7.44
		CER [%]	63.44	54.36	70.06
	TVA	WA [%]	11.38	10.65	12.38
		CER [%]	63.15	55.00	69.54
PPI	TA	WA [%]	72.09	73.13	61.38
		CER [%]	32.22	22.84	43.17
	VA	WA [%]	87.45	88.38	65.88
		CER [%]	53.31	51.70	56.44
	TVA	WA [%]	76.22	76.31	71.00
		CER [%]	37.19	28.69	39.05
PTFI	TA	WA [%]	70.81	72.95	67.24
		CER [%]	23.38	27.01	28.31
	VA	WA [%]	80.06	81.83	74.72
		CER [%]	11.86	9.80	31.20
	TVA	WA [%]	78.93	83.04	64.38
		CER [%]	19.09	18.45	29.08

**Table 6:** Categorywise word recognition performance of integration methods using Qwen-3-4B. For each category, the best WA (higher is better) and CER (lower is better) are shown in bold.

Qwen-3-4B					
Method	Feature	Metrics	Kana Syllabaries	Meaningful Words	Nonsense Strings
TI	TA	WA [%]	10.68	11.69	8.30
		CER [%]	69.88	67.35	69.22
	VA	WA [%]	11.52	13.10	9.94
		CER [%]	56.97	56.09	68.34
	TVA	WA [%]	13.04	12.79	9.96
		CER [%]	58.79	56.99	60.04
PPI	TA	WA [%]	78.38	77.00	57.81
		CER [%]	26.75	21.04	27.88
	VA	WA [%]	87.41	86.81	81.30
		CER [%]	18.52	16.85	24.39
	TVA	WA [%]	87.34	87.46	76.01
		CER [%]	22.85	24.31	31.82
PTFI	TA	WA [%]	86.30	84.89	79.31
		CER [%]	13.67	12.44	18.94
	VA	WA [%]	**88.35**	**88.69**	**82.93**
		CER [%]	**13.41**	**9.46**	**17.93**
	TVA	WA [%]	85.81	86.30	81.00
		CER [%]	15.92	14.79	20.84

## Data Availability

The publicly available ub-MOJI dataset analyzed in this study can be found in a Hugging Face repository at https://huggingface.co/datasets/kanglabs/ub-MOJI (accessed on 19 August 2026). Additional non-public recordings used in the single-character recognition experiments are not publicly available because permission for public distribution was not obtained from five participants.

## Supplementary Materials

The following supporting information can be downloaded at https://www.mdpi.com/article/10.3390/s26196044/s1, Table S1. Complete per-class precision, recall, F1-score, and support values for the visual encoder using TVA features; Table S2. Complete per-class precision, recall, F1-score, and support values for the visual encoder using VA features.
