# Supplementary material for: Investigation of Word Recognition Methods for Japanese Fingerspelling Using Large Language Models
Source: Sensors (Basel). 2026 Sep 24;26(19):6044. doi: 10.3390/s26196044 (PMC13667630; doi:10.3390/s26196044)
Supplement: Supplementary file 1 [file sensors-26-06044-s001.zip › sensors-4541546-supplementary.pdf]

Supplementary Table S1. Complete per-class precision, recall, F1-score, and support values for the visual encoder using TVA features.

Class	Precision	Recall	F1-score	Support
-	1.0000	1.0000	1.0000	3
a	0.8333	1.0000	0.9091	6
ba	0.6667	1.0000	0.8000	3
be	1.0000	1.0000	1.0000	3
bi	0.6667	0.5000	0.5714	2
bo	0.6667	1.0000	0.8000	3
bu	1.0000	1.0000	1.0000	3
da	0.5000	0.5000	0.5000	2
de	0.5000	0.5000	0.5000	2
di	1.0000	0.6667	0.8000	3
do	0.6667	0.6667	0.6667	3
du	1.0000	1.0000	1.0000	2
e	1.0000	0.8333	0.9091	6
ga	0.5000	0.6667	0.5714	3
ge	0.6667	1.0000	0.8000	2
gi	1.0000	0.6667	0.8000	3
go	1.0000	1.0000	1.0000	2
gu	1.0000	0.5000	0.6667	2
ha	0.7500	0.8000	0.7742	5
he	1.0000	1.0000	1.0000	5
hi	0.7143	0.8333	0.7692	6
ho	1.0000	1.0000	1.0000	5
hu	1.0000	1.0000	1.0000	6
i	1.0000	1.0000	1.0000	5
ka	0.7500	1.0000	0.8571	5
ke	1.0000	1.0000	1.0000	6
ki	1.0000	0.8889	0.9412	9
ko	1.0000	0.5000	0.6667	5
ku	1.0000	1.0000	1.0000	6
ma	0.8571	0.8333	0.8450	6
me	0.8571	0.8333	0.8450	6
mi	0.8571	1.0000	0.9231	5
mo	0.7500	0.7500	0.7500	4
mu	1.0000	1.0000	1.0000	6
na	1.0000	0.8333	0.9091	6
ne	1.0000	1.0000	1.0000	6
ni	0.8000	0.8333	0.8163	6
nn	0.6667	0.5000	0.5714	4
no	1.0000	1.0000	1.0000	4
nu	0.8333	1.0000	0.9091	5
o	0.6667	1.0000	0.8000	5
pa	0.5000	0.5000	0.5000	2
pe	0.6667	1.0000	0.8000	2
pi	1.0000	1.0000	1.0000	2
po	0.2500	0.0000	0.0000	2
pu	0.2500	0.5000	0.3333	2
ra	1.0000	0.8000	0.8889	5
re	1.0000	0.8333	0.9091	6
ri	0.8000	0.7500	0.7742	4
ro	1.0000	0.8000	0.8889	5
ru	1.0000	0.8333	0.9091	6
sa	0.7500	0.8000	0.7742	5
se	1.0000	1.0000	1.0000	6
si	1.0000	1.0000	1.0000	6
so	1.0000	0.6000	0.7500	5
su	1.0000	1.0000	1.0000	6
ta	1.0000	1.0000	1.0000	6

Continued on next page

Supplementary Table S1. Continued.

Class	Precision	Recall	F1-score	Support
te	1.0000	1.0000	1.0000	6
ti	1.0000	1.0000	1.0000	5
to	1.0000	0.8333	0.9091	6
tu	0.6667	0.6667	0.6667	6
u	0.6667	0.8333	0.7407	6
wa	1.0000	1.0000	1.0000	6
wo	1.0000	1.0000	1.0000	4
xtu	0.5000	1.0000	0.6667	2
xya	0.5000	1.0000	0.6667	1
xyo	1.0000	0.5000	0.6667	2
xyu	0.5000	0.0000	0.0000	2
ya	1.0000	1.0000	1.0000	5
yo	1.0000	1.0000	1.0000	5
yu	1.0000	1.0000	1.0000	6
za	1.0000	1.0000	1.0000	2
ze	1.0000	0.5000	0.6667	2
zi	1.0000	0.5000	0.6667	2
zo	1.0000	0.5000	0.6667	2
zu	1.0000	1.0000	1.0000	2
**Accuracy**			0.8644	321
**Macro avg**	0.8669	0.8402	0.8542	321
**Weighted avg**	0.9074	0.8645	0.9060	321

Supplementary Table S2. Complete per-class precision, recall, F1-score, and support values for the visual encoder using VA features.

Class	Precision	Recall	F1-score	Support
-	1.0000	1.0000	1.0000	3
a	0.8333	1.0000	0.9091	6
ba	0.6667	0.3333	0.4444	3
be	0.5000	0.3333	0.4000	3
bi	0.6667	1.0000	0.8000	2
bo	0.6667	0.6667	0.6667	3
bu	1.0000	1.0000	1.0000	3
da	1.0000	1.0000	1.0000	2
de	0.5000	0.5000	0.5000	2
di	0.4000	0.6667	0.5000	3
do	0.6667	0.6667	0.6667	3
du	1.0000	1.0000	1.0000	2
e	1.0000	1.0000	1.0000	6
ga	0.5000	0.3333	0.4000	3
ge	0.6667	1.0000	0.8000	2
gi	1.0000	0.6667	0.8000	3
go	1.0000	1.0000	1.0000	2
gu	1.0000	1.0000	1.0000	2
ha	0.7500	0.6000	0.6667	5
he	1.0000	1.0000	1.0000	5
hi	0.7143	0.8333	0.7692	6
ho	0.7500	0.6000	0.6667	5
hu	1.0000	1.0000	1.0000	6
i	1.0000	1.0000	1.0000	5
ka	0.7500	0.6000	0.6667	5
ke	1.0000	1.0000	1.0000	6
ki	1.0000	1.0000	1.0000	9
ko	1.0000	1.0000	1.0000	5
ku	1.0000	1.0000	1.0000	6
ma	0.8571	1.0000	0.9231	6
me	0.8571	1.0000	0.9231	6
mi	0.8333	1.0000	0.9091	5
mo	1.0000	1.0000	1.0000	4
mu	1.0000	1.0000	1.0000	6
na	1.0000	0.8333	0.9091	6
ne	1.0000	1.0000	1.0000	6
ni	0.8000	0.8333	0.8163	6
nn	0.4000	0.5000	0.4444	4
no	0.6667	0.5000	0.5714	4
nu	0.8333	1.0000	0.9091	5
o	0.6667	0.8000	0.7273	5
pa	0.2500	0.5000	0.3333	2
pe	0.6667	1.0000	0.8000	2
pi	1.0000	1.0000	1.0000	2
po	0.5000	0.5000	0.5000	2
pu	0.6667	1.0000	0.8000	2
ra	0.2500	0.2000	0.2222	5
re	1.0000	1.0000	1.0000	6
ri	0.8000	1.0000	0.8889	4
ro	1.0000	1.0000	1.0000	5
ru	1.0000	1.0000	1.0000	6
sa	0.7500	0.6000	0.6667	5
se	0.7500	1.0000	0.8571	6
si	0.7500	1.0000	0.8571	6
so	1.0000	0.6000	0.7500	5
su	0.8571	1.0000	0.9231	6
ta	1.0000	1.0000	1.0000	6

Continued on next page

Supplementary Table S2. Continued.

**Class**	**Precision**	**Recall**	**F1-score**	**Support**
te	1.0000	1.0000	1.0000	6
ti	1.0000	0.6000	0.7500	5
to	1.0000	1.0000	1.0000	6
tu	0.6667	0.3333	0.4444	6
u	0.6000	1.0000	0.7500	6
wa	1.0000	1.0000	1.0000	6
wo	0.6667	0.5000	0.5714	4
xtu	0.5000	1.0000	0.6667	2
xya	0.0000	0.0000	0.0000	1
xyo	0.3333	0.5000	0.4000	2
xyu	0.5000	1.0000	0.6667	2
ya	0.6667	0.8000	0.7273	5
yo	1.0000	0.4000	0.5714	5
yu	1.0000	0.6667	0.8000	6
za	1.0000	0.5000	0.6667	2
ze	1.0000	1.0000	1.0000	2
zi	1.0000	0.5000	0.6667	2
zo	1.0000	0.5000	0.6667	2
zu	1.0000	0.5000	0.6667	2
**Accuracy**			0.8287	321
**Macro avg**	0.8042	0.7825	0.7816	321
**Weighted avg**	0.8324	0.8287	0.8189	321
